# Significant association and synergistic adverse prognostic effect of podocalyxin-like protein and epidermal growth factor receptor expression in colorectal cancer

**DOI:** 10.1186/s12967-016-0882-0

**Published:** 2016-05-10

**Authors:** Anna H. Larsson, Sophie Lehn, Sakarias Wangefjord, Emelie Karnevi, Eugenia Kuteeva, Magnus Sundström, Björn Nodin, Mathias Uhlén, Jakob Eberhard, Helgi Birgisson, Karin Jirström

**Affiliations:** Division of Oncology and Pathology, Department of Clinical Sciences, Lund University, Lund, Sweden; Atlas Antibodies AB, AlbaNova University Center, Stockholm, Sweden; Department of Immunology, Genetics and Pathology, Uppsala University, Uppsala, Sweden; Science for Life Laboratory, KTH-Royal Institute of Technology, Stockholm, Sweden; Department of Surgical Sciences, Colorectal Surgery, Uppsala University, Uppsala, Sweden

**Keywords:** Podocalyxin-like, EGFR, BRAF, Colorectal cancer, Prognosis

## Abstract

**Background:**

Podocalyxin-like 1 (PODXL) is an anti-adhesive transmembrane protein that has been demonstrated to be an independent factor of poor prognosis in colorectal cancer (CRC). The gene encoding PODXL is located to chromosome 7, which also harbours the gene for the epidermal growth factor receptor (EGFR). The aim of this study was to examine the associations between PODXL and EGFR expression in CRC in vitro and in vivo.

**Methods:**

EGFR expression was analysed in tumours from three independent patient cohorts; cohort 1 (n = 533), cohort 2 (n = 259) and cohort 3 (n = 310), previously analysed for immunohistochemical PODXL expression and *KRAS* and *BRAF* mutations (cohort 1 and 3). Levels of EGFR and PODXL were determined by western blot in six different CRC cell lines.

**Results:**

High expression of PODXL was significantly associated with high EGFR expression (p < 0.001) in all three cohorts, and with *BRAF* mutation (p < 0.001) in cohort 1 and 3. High EGFR expression correlated with *BRAF* mutation (p < 0.001) in cohort 1. High EGFR expression was associated with adverse clinicopathological factors and independently predicted a reduced 5-year overall survival (OS) in cohort 1 (HR 1.77; 95 % CI 1.27–2.46), cohort 2 (HR 1.58; 95 % CI 1.05–2.38) and cohort 3 (HR 1.83; 95 % CI 1.19–2.81). The highest risk of death within 5 years was observed in patients with tumours displaying high expression of both EGFR and PODXL in cohort 1 and 3 (HR 1.97; 95 % CI 1.18–3.28 and HR 3.56; 95 % CI 1.75–7.22, respectively). Western blot analysis showed a uniform expression of PODXL and EGFR in all six examined CRC cell lines.

**Conclusions:**

The results from this study demonstrate that high expression of EGFR is an independent factor of poor prognosis in CRC. Moreover, strong links have been uncovered between expression of the recently proposed biomarker candidate PODXL with EGFR expression in CRC in vivo and in vitro, and with *BRAF* mutation in vivo. High expression of both PODXL and EGFR may also have a synergistic adverse effect on survival. These findings suggest a potential functional link in CRC between PODXL, EGFR and BRAF, all originating from chromosome 7, which may be highly relevant in the clinical setting and therefore merit future in-depth study.

**Electronic supplementary material:**

The online version of this article (doi:10.1186/s12967-016-0882-0) contains supplementary material, which is available to authorized users.

## Background

Podocalyxin-like protein (PODXL) is a transmembrane glycoprotein and member of the CD34 family [1]. PODXL regulates cell adhesion and is normally expressed by podocytes of the kidney, vascular endothelial cells and hematopoietic progenitor cells [2–4]. Moreover, PODXL has been shown to be overexpressed in a variety of malignancies, and associated with a more aggressive tumour phenotype and poor prognosis in breast, prostate, colorectal, ovarian, renal, pancreatic and bladder cancer, as well as in glioblastoma and astrocytoma [5–14]. The adverse prognostic role of PODXL expression in colorectal cancer (CRC) was first described only recently [7], and has since then been validated in several independent studies [15–17].

The exact mechanisms behind the role of PODXL in tumourigenesis are still unknown, but it has been demonstrated to be involved in epithelial-mesenchymal transition (EMT), a process in which epithelial cells obtain mesenchymal properties leading to increased migration, invasiveness and resistance to apoptosis [18]. In two recent studies, knockdown of *PODXL* in breast cancer cell lines resulted in impaired primary tumour growth and metastasis [19, 20].

The gene encoding PODXL is located to chromosome 7, which also harbours several other genes with important implications in CRC, e.g. the genes encoding the epidermal growth factor receptor (EGFR) and v-Raf murine sarcoma viral oncogene homolog B (BRAF). Of note, the *PODXL* and *BRAF* genes are located right next to each other at 7q32-33 and 7q34.

EGFR is a transmembrane receptor tyrosine kinase that plays an important role in CRC initiation and progression through the RAS–RAF–MEK- MAPK and the PI3K–PTEN–Akt signalling pathways. Overexpression of EGFR has been reported in 25–75 % of CRC [21].

The clinical significance of EGFR overexpression in CRC remains unclear. Whereas several studies have demonstrated a link between high EGFR expression and poor prognosis [22–26], other studies have not found EGFR expression to correlate with an adverse outcome [22, 27, 28]. However, due to its role in the progression of CRC, EGFR has become an interesting target for antitumoural therapy, and monoclonal anti-EGFR antibodies cetuximab and panitumumab are widely used in metastatic CRC [29].

The aim of this study was to investigate the relationship between PODXL and EGFR expression in CRC in vivo and in vitro. To this end, immunohistochemical expression of the proteins was compared in tumours from three different patient cohorts, and western blot analysis was performed on six different CRC cell lines.

## Methods

### Patients

Cohort 1 encompasses tumours from incident CRC cases in the population-based, prospective cohort Malmö Diet and Cancer Study (MDCS). Until end of follow-up 31 December 2008, 626 incident cases of CRC had been registered in the study population, and tumour tissue for tissue microarray (TMA) was available from 557 patients. The cohort has been described previously [7, 30, 31].

Cohort 2 is a consecutive, retrospective cohort comprising all patients who underwent surgery for CRC at Skåne University Hospital in Malmö, Sweden between 1 January 1990 and 31 December 1991, for whom archival tumour tissue was available (n = 270). The cohort has been described previously [15, 32, 33].

Cohort 3 consists of 337 patients who were surgically treated for CRC at the Central District Hospital in Västerås, Sweden between August 2000 and December 2003. TMAs were constructed from 320 patients. The cohort has been described previously [15, 32, 34].

Patient and tumour characteristics in the different cohorts are summarized in Additional file 1.

Approvals for the study were obtained from the Ethics Committees at Lund University (ref 51/90, 530/08 and 445/2007) and Uppsala University (ref 00/001).

### PODXL and EGFR immunohistochemistry and evaluation

Formalin-fixed, paraffin-embedded CRC tissue blocks were used to construct TMAs as previously described [7], and immunohistochemical staining was performed on 4 μm TMA-sections.

PODXL expression has previously been analysed in all three cohorts [7, 15]. In brief, the affinity-purified polyclonal anti-PODXL antibody HPA 2110 (Atlas Antibodies, Stockholm, Sweden), diluted 1:250 was used, and staining was performed in an Autostainer Plus (Dako, Glostrup, Denmark) after automated pre-treatment with the PT-link system (Dako). PODXL expression was denoted as negative (0), weak cytoplasmic staining (1), moderate cytoplasmic staining (2), distinct membranous staining in ≤50 % of tumour cells (3) and distinct membranous staining in > 50 % of tumour cells (4), as previously described [7]. Based on the presence or absence of membranous staining, PODXL expression was dichotomised into low (0–2) or high (3–4).

For immunohistochemical analysis of EGFR, TMA-sections were automatically pre-treated using the PT-link system (DAKO, Glostrup, Denmark) and then stained in an Autostainer Plus (DAKO, Glostrup, Denmark) with the monoclonal antibody 31G7 (Zymed Laboratories Inc, San Francisco, CA, USA, diluted 1:25). For validatory purposes, TMAs from cohort 1 were also stained with the primary anti-EGFR antibody 3C6 (Ventana Medical Systems, Tucson, AZ, USA), stained in a BenchMark ULTRA (Ventana Medical Systems). Antigen retrieval was performed with protease1 (Ventana Medical Systems) for 8 min and antibody incubation time was 32 min in 36 °C. EGFR expression was determined by the intensity (0–3) of membranous staining in tumour cells in line with the scoring protocol proposed by Goldstein [21]. A score of 0–1 was considered low EGFR expression, and 2–3 indicated high EGFR expression.

Assessment of PODXL and EGFR expression was performed in the same way for all three cohorts by two independent observers (AL and KJ). Interobserver differences were discussed in order to reach consensus.

### Analysis of *KRAS* and *BRAF* mutation status

The PyroMark Q24 system (Qiagen GmbH, Hilden, Germany) was used for pyrosequencing analysis of *KRAS* and *BRAF* mutations in DNA from 1 mm formalin-fixed, paraffin-embedded or fresh frozen tumour tissue cores taken from areas with >90 % tumour cells, as previously described [35]. In brief, DNA was isolated from tumour tissue using QIAamp MinElute spin columns (Qiagen) and DNA regions of interest were PCR-amplified (Veriti 96-Well Fast Thermal Cycler, Applied Biosystems Inc., Foster City, CA, USA).

Detection of mutations in *KRAS* codons 12 and 13 was performed using Therascreen KRAS Pyro Kit (Qiagen). Analysis of *BRAF* mutation hotspots in codons 600 and 601 was performed using previously published PCR primers (Richman, JCO 2009) and a novel BRAF sequencing primer (5′-TGATTTTGGTCTAGCTACA-3′) which was designed using the PyroMark Assay Design 2.0 software (Qiagen). All samples with a potential low-level mutation were re-analysed.

### Cell culture

CRC cell lines Caco-2, RKO, SW480, SW620, HCT-116 and HT-29 were used and maintained in a humified atmosphere at 37 °C and 5 % carbon dioxide/95 % air. Caco-2 and RKO cell lines were grown in EMEM supplemented with 2 mM l-glutamine, 10 % FBS (fetal bovine serum) and 1XPEST (penicillin 90 IU/ml and streptomycin 90 µg/ml). SW480 and SW620 were maintained in DMEM with 4 mM l-glutamine, 4500 mg/L glucose, 1 mM sodium pyruvate, 10 % FBS and 1XPEST, while HCT-116 and HT-29 were grown in McCoy’s 5A medium supplemented with 1.5 mM l-glutamine, 2.2 g/L sodium bicarbonate, 10 % FBS and 1XPEST.

### Western blot

The levels of EGFR and PODXL in cell lines were determined by western blot. To check for basal levels of the proteins, cells were harvested when still sub-confluent. For western blot analysis, cells were trypsinised and washed in PBS. Cell pellets were kept at −80 °C for at least 24 h after which they were lysed in RIPA buffer (Sigma-Aldrich Co, St Louis, MO, cat #R0278: 150 mM NaCl, 1.0 % IGEPAL^®^ CA-630, 0.5 % sodium deoxycholate, 0.1 % SDS, 50 mM Tris, pH 8.0), supplemented with protease inhibitors and phos-STOP (Roche, Basel, Switzerland). Lysates were centrifuged at 4 °C for 10 min at 6200 g and supernatants collected. Protein concentrations were determined using the BCA Protein Assay (Thermo Scientific, Rockford, IL). Twenty µg of protein was separated on 4–15 % graded Mini-PROTEAN^®^ TGX™ precast gels (Bio-Rad Laboratories Inc. Hercules, CA). For PODXL blots, an XCell *SureLock*^®^ Mini-Cell (Thermo Scientific, Rockford, IL) wet transfer system was used (25 V for 2 h) with a transfer buffer containing 25 mM Tris base, 192 mM glycine, 20 % ethanol at a pH of 8.3. PVDF membranes were activated for 30 s in 99.5 % ethanol before transfer sandwich assembly. For EGFR blots, the Trans-Blot^®^ Turbo™ Mini PVDF Transfer packs were used together with the Trans-Blot^®^ Turbo™ Transfer System (Bio-Rad Laboratories Inc. Hercules, CA). Membranes were subsequently blocked with 5 % non-fat dry milk in TBS-tween 0.1 % and probed with the following antibodies overnight: EGFR (Cell Signaling Inc., Danvers, MA, cat#4267, dilution 1:1000), PODXL (Atlas Antibodies, cat#HPA002110, 1:500) and actin (Santa Cruz Biotechnology Inc., Dallas, TX, cat#sc-1616, 1:1000). Three sets of lysates were prepared and blotted for every cell line, and one representative experiment is shown.

### Cell pellet array and immunocytochemistry

Cell lines were trypsinised and washed in PBS. Subsequently, cell pellets were fixed in formalin for at least 24 h followed by staining with Mayer’s haematoxylin for 5 min. Cells were washed once in PBS and dehydrated in graded ethanol series after which cell pellets were washed in molten paraffin several times. Cell pellets were arrayed in duplicate or triplicate 1.0 mm cores using a semi-automated arraying device (TMArrayer; Pathology Devices, Inc., Westminster, MD). Immunohistochemistry was performed on 5 µm sections using the same antibodies as for western blot with the following dilutions: EGFR, 1:100 and PODXL, 1:500. Images were captured at 20X using the cellSens entry software (version 1.8, Olympus).

### Statistical analysis

The Chi square test was applied for comparison of PODXL expression with EGFR expression and molecular characteristics, and for comparison of EGFR expression with established prognostic clinicopathological factors. Kaplan–Meier analysis and log rank test were applied to illustrate differences in 5-year OS according to PODXL and EGFR expression. Cox’s proportional hazards regression was used for estimation of hazard ratios (HR) for death from CRC within 5 years, according to PODXL and EGFR expression in both univariable and multivariable analysis adjusted for age, sex, T-, N-, M-stage, differentiation grade and vascular invasion. Co-variables were entered into the multivariable analysis using backward selection where a p value of 0.05 decided entry and a p-value of 0.10 was used for removal. All tests were two-sided. A p value of 0.05 was considered significant. All statistical analyses were performed using SPSS version 20.0 (SPSS Inc, Chicago, IL).

## Results

### Overexpression of PODXL is associated with EGFR expression and *BRAF* mutation in colorectal cancer

**Fig. 1:**
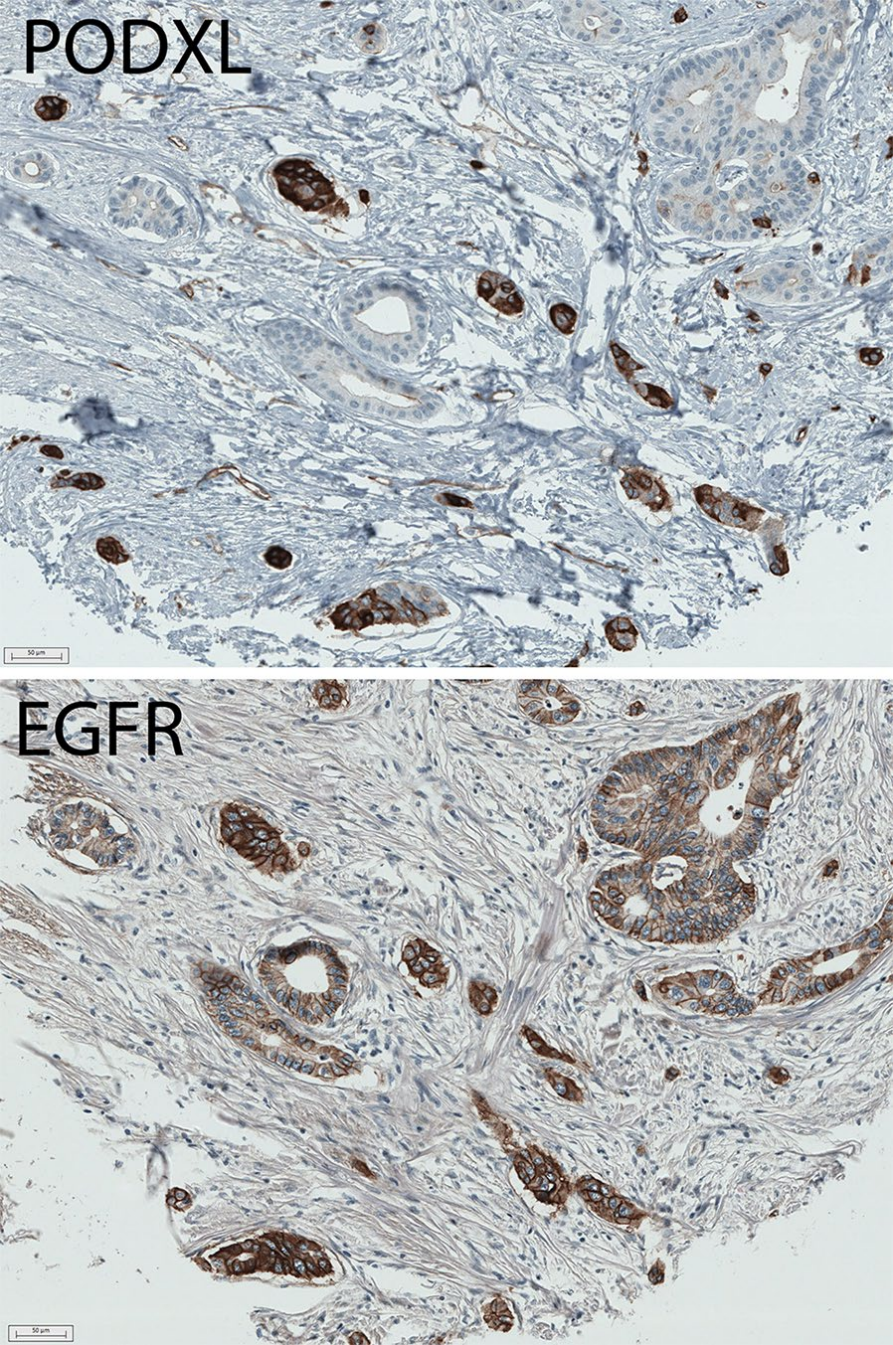
Sample immunohistochemical images. Immunohistochemical image of a colorectal tumour with high expression of both EGFR and PODXL. Note the subset of infiltrative cells with particularly strong membranous expression of both proteins

EGFR could be analysed in 533/626 (85.1 %) cases in cohort 1, 259/270 (95.9 %) cases in cohort 2, and 310/337 (92.0 %) cases in cohort 3. Sample immunohistochemical images are shown in Fig. 1.

The intercorrelation between PODXL and other investigative factors are shown in Table 1. High expression of PODXL was significantly associated with high EGFR expression (p < 0.001) in all three cohorts, and with *BRAF* mutation (p < 0.001) and MSI (p < 0.001 and p = 0.021 respectively) in cohort 1 and 3. There was no significant correlation between PODXL expression and *KRAS* mutation (cohort 1 and 3). Information on *BRAF* status, MSI and *KRAS* mutation was not available in cohort 2.

**Table 1:** Associations between PODXL expression and EGFR expression, *BRAF,* *KRAS* mutations and MSI status in colorectal cancer

PODXL	Cohort 1					*p value*	Cohort 2					*p value*	Cohort 3					*p value*
	0	1	2	3	4		0	1	2	3	4		0	1	2	3	4	
N (%)	268 (50.0)	186 (34.7)	10 (1.9)	71 (13.2)	1 (0.2)		137 (50.7)	67 (24.8)	31 (11.5)	25 (9.3)	0 (0.0)		197 (56.3)	65 (18.6)	29 (8.3)	22 (6.3)	3 (0.9)	
EGFR																		
Low	225 (87.5)	141 (77.9)	7 (70.0)	34 (48.6)	0 (0.0)	<*0.001*	106 (80.3)	55 (84.6)	22 (73.3)	12 (50.0)	0 (0.0)	*0.008*	171 (89.5)	55 (85.9)	21 (72.4)	10 (47.6)	0 (0.0)	<*0.001*
High	32 (12.5)	40 (22.1)	3 (30.0)	36 (51.4)	1 (100.0)		26 (19.7)	10 (15.4)	8 (26.7)	12 (50.0)	0 (0.0)		20 (10.5)	9 (14.1)	8 (27.6)	11 (52.4)	3 (100.0)	
*Missing*	*11*	*5*		*1*			*5*	*2*	*1*	*1*			*6*	*1*		*1*		
*BRAF* status																		
Wt	227 (90.8)	153 (85.5)	4 (40.0)	45 (70.3)	0 (0.0)	<*0.001*	N/A	N/A	N/A	N/A	N/A		123 (89.8)	33 (78.6)	12 (60.0)	13 (81.3)	0 (0.0)	<*0.001*
Mutated	23 (9.2)	26 (14.5)	6 (60.0)	19 (29.7)	1 (100.0)		N/A	N/A	N/A	N/A	N/A		14 (10.2)	9 (21.4)	8 (40.0)	3 (18.7)	2 (100.0)	
*Missing*	*18*	*7*		*7*									*60*	*23*	*9*	*6*	*1*	
*KRAS* status																		
Wt	153 (61.0)	118 (65.9)	9 (90.0)	41 (64.1)	1 (100.0)	*0.312*	N/A	N/A	N/A	N/A	N/A		75 (58.1)	29 (70.7)	17 (85.0)	8 (57.1)	2 (100.0)	*0.095*
Mutated	98 (39.0)	61 (34.1)	1 (10.0)	23 (35.9)	0 (0.0)		N/A	N/A	N/A	N/A	N/A		54 (41.9)	12 (29.3)	3 (15.0)	6 (42.9)	0 (0.0)	
*Missing*	*17*	*7*		*7*									*68*	*24*	*9*	*8*	*1*	
MSI status																		
MSS	227 (88.7)	146 (84.9)	0 (0.0)	52 (80.0)	1 (100.0)	<*0.001*	N/A	N/A	N/A	N/A	N/A		170 (86.7)	51 (81.0)	20 (69.0)	22 (100.0)	2 (66.7)	*0.021*
MSI	29 (11.3)	26 (15.1)	9 (100.0)	13 (20.0)	0 (0.0)		N/A	N/A	N/A	N/A	N/A		26 (13.3)	12 (19.0)	9 (31.0)	0 (0.0)	1 (33.3)	
*Missing*	*12*	*14*	*1*	*6*									*1*	*2*				

Associations of EGFR expression with established clinicopathological and investigative factors are shown in Table 2. In cohort 1 and 3, high EGFR expression was significantly associated with more advanced T-, N-, M-stage, low differentiation grade and vascular invasion. In cohort 2, there was a significant association between high EGFR expression and M-stage. Furthermore, high EGFR expression was significantly associated with *BRAF* mutation in cohort 1 (p < 0.001).

**Table 2:** Associations of EGFR status with clinicopathological characteristics in three independent CRC patient cohorts

EGFR	Cohort 1		*p* *value*	Cohort 2		*p* *value*	Cohort 3		*p* *value*
	Low	High		Low	High		Low	High	
N (%)	419 (78.6)	114 (21.4)		202 (78.0)	57 (21.1)		259 (83.5)	51 (16.5)	
Age									
Mean, median	70.6, 71.4	69.9, 71.3	*0.439*	72.6, 73.5	70.7, 73.1	*0.401*	71.1, 73.0	71.5, 74.0	*0.977*
Range	51.3–85.6	49.8–83.5		37.6–92.1	37.8–93.3		36.0–94.0	50.0–87.0	
Sex									
Female	219 (52.3)	62 (54.4)	*0.688*	98 (48.5)	34 (59.6)	*0.138*	129 (49.8)	29 (56.9)	*0.358*
Male	200 (47.7)	52 (45.6)		104 (51.5)	23 (40.4)		130 (50.2)	22 (43.1)	
Location									
Colon	258 (61.9)	76 (66.7)	*0.348*	161 (79.7)	48 (85.7)	*0.311*	177 (68.3)	30 (58.8)	*0.188*
Rectum	159 (38.1)	38 (33.3)		41 (20.3)	8 (14.3)		82 (31.7)	21 (41.2)	
*Missing*	*2*								
T-stage									
T1	43 (10.9)	1 (0.9)	<*0.001*	14 (7.1)	4 (7.1)	*0.432*	11 (4.2)	0 (0.0)	*0.002*
T2	55 (13.9)	7 (6.2)		53 (26.8)	11 (19.6)		38 (14.7)	1 (2.0)	
T3	249 (62.9)	75 (67.0)		107 (54.0)	33 (58.9)		169 (65.3)	37 (72.5)	
T4	49 (12.4)	29 (25.9)		24 (14.3)	8 (14.3)		41 (15.8)	13 (25.5)	
*Missing*	*23*	*2*		*4*	*1*				
N-stage									
N0	232 (61.7)	48 (43.2)	*0.002*	129 (64.8)	33 (61.1)	*0.331*	164 (63.39	18 (35.3)	<*0.001*
N1	87 (23.1)	34 (30.6)		50 (25.1)	12 (22.2)		53 (20.5)	9 (17.6)	
N2	57 (15.2)	29 (26.1)		20 (10.1)	9 (16.7)		42 (16.2)	24 (47.1)	
*Missing*	*43*	*3*		*3*	*3*				
M-stage									
M0	349 (84.5)	82 (72.6)	*0.003*	180 (89.6)	40 (72.7)	*0.002*	232 (89.6)	39 (76.5)	*0.010*
M1	64 (15.5)	31 (27.4)		21 (10.4)	15 (27.3)		27 (10.4)	12 (23.5)	
*Missing*	*6*	*1*		*1*	*2*				
Diff. grade									
High	28 (6.8)	4 (3.6)	<*0.001*	16 (7.9)	5 (8.8)	*0.402*	8 (3.1)	1 (2.0)	*0.006*
Intermediate	310 (75.4)	62 (55.4)		138 (68.3)	34 (59.6)		202 (78.0)	31 (60.8)	
Low	73 (17.8)	46 (41.1)		48 (23.8)	18 (31.6)		49 (18.9)	19 (37.3)	
*Missing*	*8*	*2*							
Vasc. invasion									
No	125 (53.2)	27 (34.2)	*0.003*	108 (54.8)	24 (44.4)	*0.177*	233 (90.0)	38 (74.5)	*0.002*
Yes	110 (46.8)	52 (65.8)		89 (45.2)	30 (55.6)		26 (10.0)	13 (25.5)	
*Missing*	*184*	*35*		*5*	*3*				
MSI status									
MSS	333 (85.4)	85 (81.0)	*0.266*	N/A	N/A		214 (82.9)	47 (92.2)	*0.098*
MSI	57 (14.6)	20 (19.0)		N/A	N/A		44 (17.1)	4 (7.8)	
*Missing*	*29*	*9*					*1*		
*KRAS* status									
Wild-type	253 (63.9)	66 (61.1)	*0.596*	N/A	N/A		108 (63.5)	24 (70.6)	*0.433*
Mutated	119 (36.1)	42 (38.9)		N/A	N/A		62 (36.5)	10 (29.4)	
*Missing*	*23*	*6*					*89*	*17*	
*BRAF* status									
Wild-type	348 (88.1)	78 (72.2)	<*0.001*	N/A	N/A		152	27	*0.147*
Mutated	47 (11.9)	30 (27.8)		N/A	N/A		27	9	
*Missing*	*24*	*6*					*80*	*15*	

Kruskal–Wallis or Mann–Whitney U test applied for continuous variables

*MSI* microsatellite instability; *MSS* microsatellite stable

As further shown in Additional file 2, there was a very good correlation between EGFR protein expression assessed by both antibodies in cohort 1.

### Overexpression of EGFR is associated with a poor prognosis, in particular in combination with PODXL overexpression

**Fig. 2:**
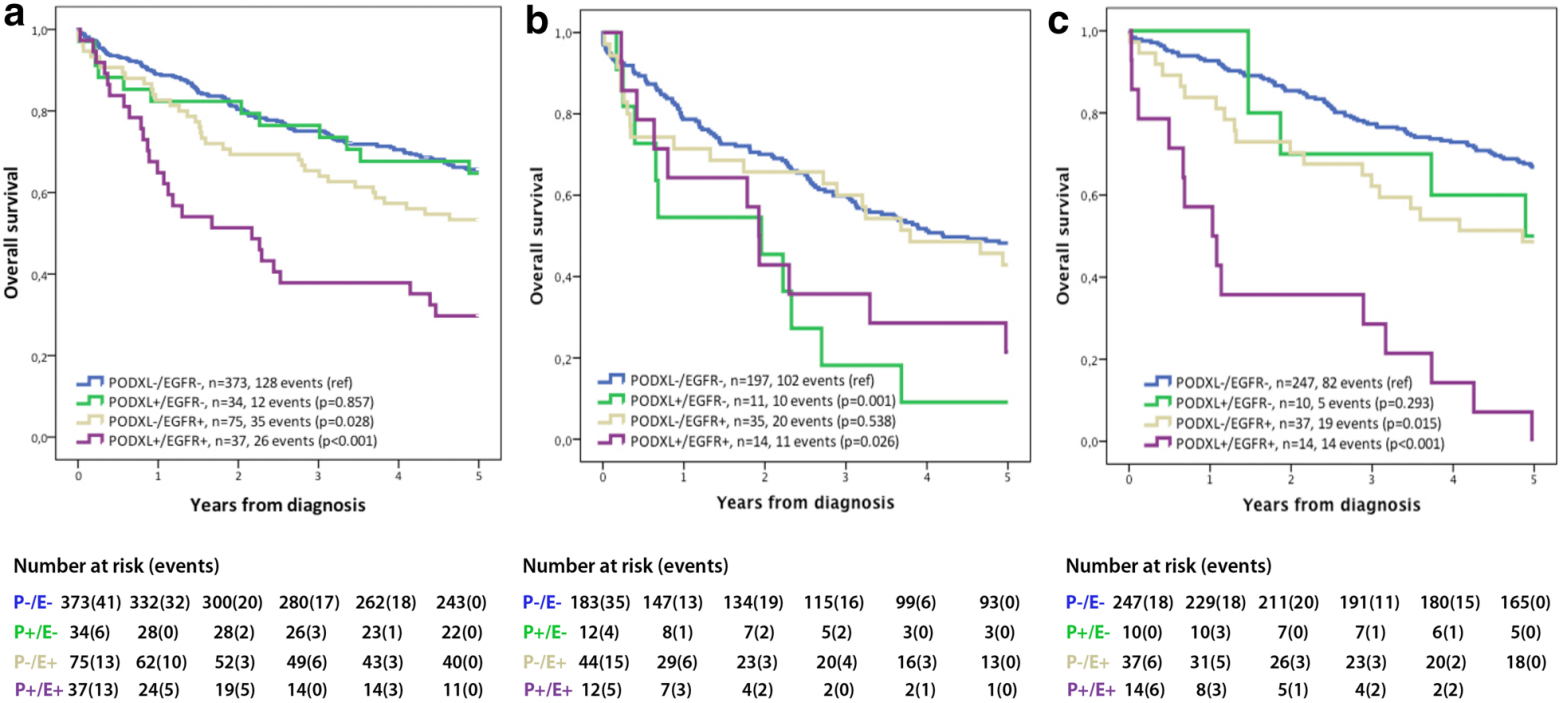
Kaplan–Meier analysis. Kaplan-Meier estimates of 5-year OS according to combinations of PODXL and EGFR expression in cohort 1 (**a**), cohort 2 (**b**) and cohort 3 (**c**). Log rank p values correspond to pairwise comparisons of colorectal tumours with low expression of PODXL and EGFR with the other strata

Kaplan–Meier analysis showed that high EGFR expression, in particular in combination with high PODXL expression, correlated with a reduced overall survival in all three cohorts (Fig. 2). As shown in Table 3, high EGFR expression was an independent predictor of a reduced 5-year OS in cohort 1 (HR 1.77; 95 % CI 1.27–2.46), cohort 2 (HR 1.58; 95 % CI 1.05–2.38) and cohort 3 (HR 1.83; 95 % CI 1.19–2.81). The highest risk of death within 5 years was observed in patients with tumours displaying high expression of both EGFR and PODXL in cohort 1 and 3 (unadjusted HR 1.97; 95 % CI 1.18–3.28 and HR 3.56; 95 % CI 1.75–7.22, respectively), remaining significant in adjusted analysis in cohort 3 (HR 3.71; 95 % CI 1.23–11.20). P values for term of interaction between EGFR and PODXL in cohort 1, 2 and 3 were 0.114, 0.690 and 0.147 respectively. In cohort 2, high PODXL expression was an independent prognostic factor in patients with tumours displaying low but not high EGFR expression (unadjusted HR 2.02; 95 % CI 1.02–4.02) and adjusted HR 2.59; 95 % CI 1.26–5.31).

**Table 3:** Cox regression analysis of relative risks of death within 5 years according to EGFR and PODXL expression in colorectal cancer

	Cohort 1			Cohort 2			Cohort 3		
	HR (95 % CI)	HR (95 % CI)	N (events)	HR (95 % CI)	HR (95 % CI)	N (events)	HR (95 % CI)	HR (95 % CI)	N (events)
	Unadjusted	Adjusted		Unadjusted	Adjusted		Unadjusted	Adjusted	
All									
EGFR low	1.00	1.00	419 (146)	1.00	1.00	202 (101)	1.00	1.00	259 (88)
EGFR high	1.82 (1.35–2.46)	1.77 (1.27–2.46)	114 (61)	1.98 (1.39–2.84)	1.58 (1.05–2.38)	57 (43)	2.60 (1.74–3.89)	1.83 (1.19–2.81)	51 (33)
All									
PODXL low	1.00	1.00	464 (168)	1.00	1.00	235 (123)	1.00	1.00	291 (103)
PODXL high	1.73 (1.21–2.46)	1.16 (0.77–1.73)	72 (38)	2.27 (1.43–3.62)	1.91 (1.11–3.27)	25 (21)	3.45 (2.13–5.58)	1.23 (0.68–2.22)	25 (20)
EGFR low									
PODXL low	1.00	1.00	373 (128)	1.00	1.00	183 (89)	1.00	1.00	247 (82)
PODXL high	1.06 (0.58–1.91)	0.87 (0.45–1.66)	34 (12)	2.02 (1.02–4.02)	2.59 (1.26–5.31)	12 (9)	1.62 (0.65–3.99)	0.74 (0.28–1.95)	10 (5)
EGFR high									
PODXL low	1.00	1.00	75 (35)	1.00	1.00	44 (31)	1.00	1.00	37 (19)
PODXL high	1.97 (1.18–3.28)	1.38 (0.71–2.68)	37 (26)	1.60 (0.80–3.21)	1.69 (0.74–3.84)	12 (11)	3.56 (1.75–7.22)	3.71 (1.23–11.20)	14 (14)

Cohort 1 and 2 adjusted for age at surgery, sex, PODXL, EGFR, T-, N-, M-stage, differentiation grade and vascular invasion in multivariable analysis

Cohort 3 adjusted for age at surgery, sex, PODXL, EGFR, T-, N-, M-stage, differentiation grade, vascular invasion and neural invasion in multivariable analysis

### PODXL and EGFR levels in colorectal cancer cell lines

**Fig. 3:**
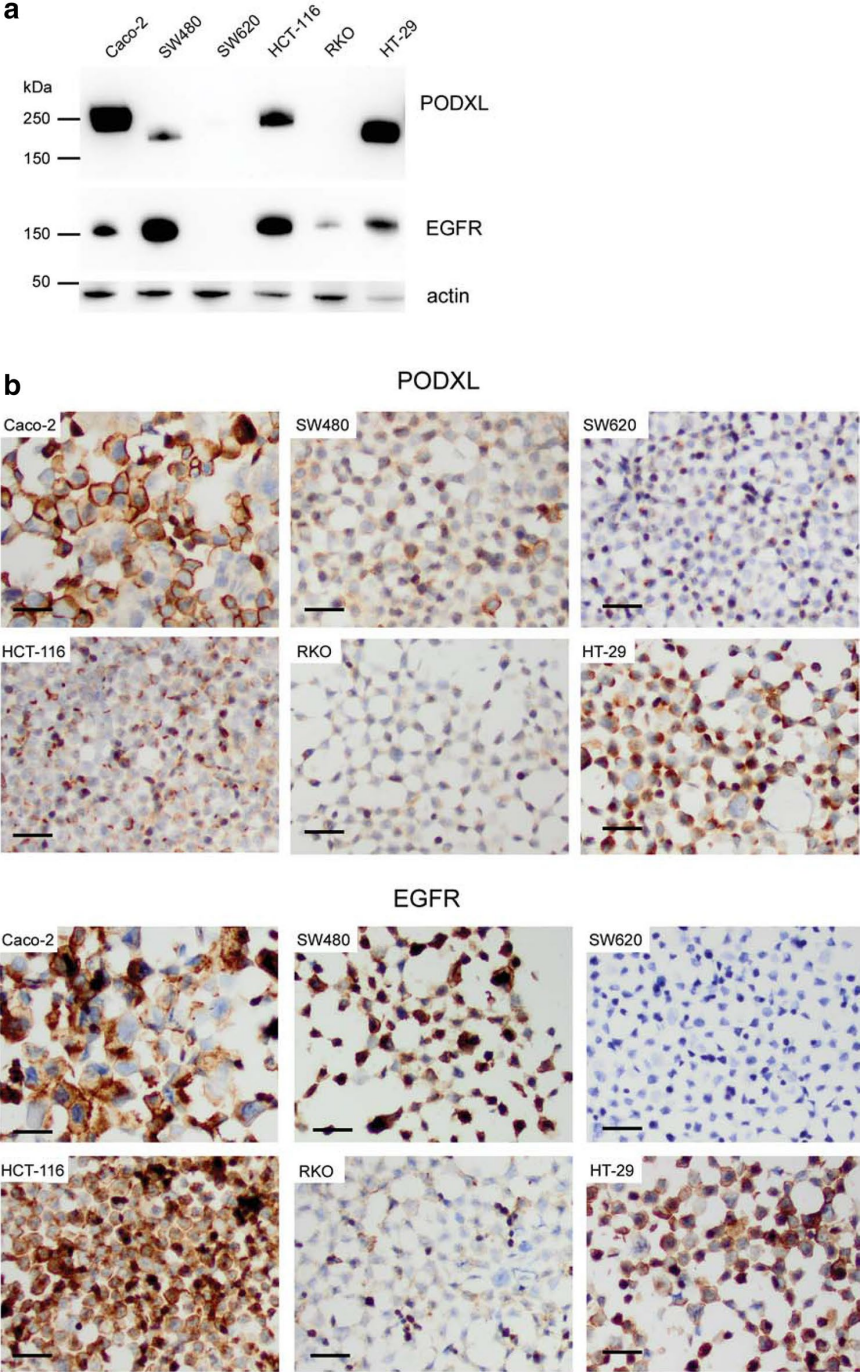
Western blot and immunocytochemical analysis of EGFR and PODXL in CRC cells. **a** Western blot and **b** immunocytochemical analysis of PODXL and EGFR protein levels in six different CRC cell lines; Caco-2, SW480, SW620, HCT-116, RKO and HT-29

As shown in Fig. 3, western blot analysis demonstrated that all cell lines with expression of PODXL also expressed EGFR, whereas the cell lines without PODXL expression did not. In cell lines derived from the same patient, EGFR and PODXL were expressed in the primary tumour cell line (SW480), but not in the metastatic derivative (SW620).

## Discussion

Chromosome 7 harbours several genes of importance in CRC, e.g. *EGFR*, *PODXL* and *BRAF* [36]. The results from the present study, based on analyses of tumours from more than 1100 patients, demonstrate, for the first time, strong significant associations between high protein expression of PODXL and EGFR in CRC. In the two largest examined cohorts, where data on *BRAF* mutation and MSI status was available for the majority of cases, PODXL expression was also found to correlate with *BRAF* mutation and MSI. The correlation between PODXL and EGFR was further demonstrated in vitro, with the proteins being uniformly expressed in six different CRC cell lines.

Moreover, the results from this study, based on immunohistochemical analysis of tumours from three independent patient cohorts, demonstrate that high EGFR protein expression is an independent negative prognostic factor in CRC patients. Despite diverging results in the literature regarding the prognostic significance of EGFR, these findings are in line with several previous studies, wherein EGFR protein expression has been associated with advanced disease stage [37, 38] and poor survival [39–42], hence adding further weight to the feasibility of EGFR as a negative prognostic biomarker in CRC.

Of note, the herein used anti-EGFR antibody was validated against another antibody, showing high concordance. These antibodies from Zymed and Ventana have also been demonstrated to perform better than others [43].

Expression of PODXL has previously been shown to be an independent adverse prognostic factor in all three herein investigated cohorts [7, 15]. In cohort 1 and 3, the worst prognosis was seen in patients with tumours displaying high expression of both EGFR and PODXL. This observation indicates that there may be a synergistic adverse prognostic effect of PODXL and EGFR, even if there was no significant interaction. Moreover, the results from cohort 2 differed somewhat in that high EGFR expression was an independent prognostic factor in PODXL low, but not high, tumours. Of note, in cohort 2, with exception for M-stage, there were no significant associations of EGFR expression with established unfavourable clinicopathological factors, whereas in cohort 1 and 3, high EGFR expression was significantly associated with more advanced T-, N- and M-stage, low differentiation grade and vascular invasion. In contrast to EGFR, high PODXL expression was found to correlate with more advanced N-stage, low differentiation grade and vascular invasion in cohort 2 [15].

Management of CRC has improved immensely over the past decades due to refined surgical techniques and optimal use of chemotherapy. The introduction of targeted therapies including bevacizumab and anti-EGFR antibodies has further improved outcome for patients with metastatic CRC. Disease stage is still the strongest prognostic factor, however, in this era of personalised medicine, new classification systems based on prognostic and predictive markers are needed to select the most efficient treatment for patients. In recent years, different research groups have proposed new classification systems based on gene expression in colorectal tumours [44–46]. Independently, all groups have indentified one subtype that is associated with EMT, poor differentiation and unfavorable prognosis. EMT is considered a critical step in the progression to metastasis, and PODXL plays an important role in this process [18].

*KRAS, NRAS* and *BRAF* mutation status is used as predictive markers for response to treatment with monoclonal anti-EGFR antibodies. However, only approximately 40 % of CRC patients with tumours wild-type for *KRAS, NRAS* and *BRAF* benefit from such therapy [47–50], and patients who initially respond eventually become resistant to these drugs. Several potential mechanisms of acquired resistance to anti-EGFR drugs have been proposed, one of them being EMT. Evidence suggests that EGFR signalling can trigger EMT [51], but once EMT is established, signalling associated with EGFR activation is reduced [52]. Moreover, studies on non-small cell lung cancer (NSCLC) and CRC cell lines have shown that tumour cells that have undergone EMT are much less sensitive to anti-EGFR treatment [53, 54]. In a study by Buck et al., CRC cell lines derived from the same patient showed epithelial characteristics and sensitivity to EGFR tyrosine kinase inhibitor erlotinib in cells from the primary tumour, whereas tumour cells from the liver metastasis exhibited a mesenchymal phenotype and were not sensitive to erlotinib [54]. Interestingly, in our study, using the same cell lines, EGFR and PODXL were expressed in cells from the primary tumour, but not in the metastatic cell line. Thus, EMT, and possibly PODXL, may have a role in resistance to anti-EGFR drugs by activating alternative signalling pathways.

Moreover, previous in vitro studies have shown that expression of PODXL leads to recruitment of the Na +/H + Exchanger Regulatory Factor (NHERF) proteins to the apical domain of the epithelial cell [55]. NHERF-1 in turn has been shown to stabilise EGFR at the cell surface to restrict receptor downregulation, thus enhancing EGFR signalling [56]. Based on these results it would be of interest to investigate whether PODXL may affect the response to monoclonal anti-EGFR antibody therapy by limiting the downregulation of EGFR receptors through NHERF-1.

## Conclusions

The results from this study demonstrate that high expression of EGFR is an independent factor of poor prognosis in CRC. Moreover, strong links have been uncovered between expression of the recently proposed biomarker candidate PODXL with EGFR expression in CRC in vivo and in vitro, and with *BRAF* mutation in vivo. High expression of both PODXL and EGFR may also have a synergistic adverse effect on survival. Taken together, these findings suggest a functional link in CRC between PODXL, EGFR and BRAF, all originating from chromosome 7. Future in-depth studies are warranted to further elucidate the mechanistic basis underlying these observations, which may be highly relevant in the clinical setting.

## Additional files

10.1186/s12967-016-0882-0 Patient and tumour characteristics in the three cohorts.
10.1186/s12967-016-0882-0 Comparison of EGFR protein expression in CRC assessed by two different antibodies in cohort 1.
